# Engineering of Optimized Hydrogel Formulations for Cartilage Repair

**DOI:** 10.3390/polym13091526

**Published:** 2021-05-10

**Authors:** Yao Fu, Bram Zoetebier, Sanne Both, Pieter J. Dijkstra, Marcel Karperien

**Affiliations:** Developmental BioEngineering, TechMed Centre, Faculty of Science and Technology, University of Twente, P.O. Box 217, 7500 AE Enschede, The Netherlands; fuyao1024@gmail.com (Y.F.); s.k.both@utwente.nl (S.B.); p.j.dijkstra@utwente.nl (P.J.D.)

**Keywords:** hydrogel scaffolds, cartilage regeneration, mechanical properties, stem cells, matrix formation

## Abstract

The ideal scaffold for cartilage regeneration is expected to provide adequate mechanical strength, controlled degradability, adhesion, and integration with the surrounding native tissue. As it does this, it mimics natural ECMs functions, which allow for nutrient diffusion and promote cell survival and differentiation. Injectable hydrogels based on tyramine (TA)-functionalized hyaluronic acid (HA) and dextran (Dex) are a promising approach for cartilage regeneration. The properties of the hydrogels used in this study were adjusted by varying polymer concentrations and ratios. To investigate the changes in properties and their effects on cellular behavior and cartilage matrix formation, different ratios of HA- and dextran-based hybrid hydrogels at both 5 and 10% *w*/*v* were prepared using a designed mold to control generation. The results indicated that the incorporation of chondrocytes in the hydrogels decreased their mechanical properties. However, rheological and compression analysis indicated that 5% *w*/*v* hydrogels laden with cells exhibit a significant increase in mechanical properties after 21 days when the constructs are cultured in a chondrogenic differentiation medium. Moreover, compared to the 10% *w*/*v* hydrogels, the 5% *w*/*v* hybrid hydrogels increased the deposition of the cartilage matrix, especially in constructs with a higher Dex–TA content. These results indicated that 5% *w*/*v* hybrid hydrogels with 25% HA–TA and 75% Dex–TA have a high potential as injectable scaffolds for cartilage tissue regeneration.

## 1. Introduction

Articular cartilage is a firm, smooth, viscoelastic padding at the ends of bones that ensure smooth, frictionless, and pain-free joint movement [1,2]. Articular cartilage tissue is highly hydrated and consists of approximately 70% water and 30% extracellular matrix (ECM) [3]. The structure and function of articular cartilage depends on the molecular composition of its ECM, mainly collagens and proteoglycans [4]. Cartilage has a limited capacity for self-repair due to its avascular nature and the low mitotic activity of chondrocytes [5]. Without proper treatment, damaged articular cartilage will deform, causing chronic pain and joint disability.

Over the past decades, several pharmacological and regenerative therapies have been developed [6]. An ideal scaffold for cartilage regeneration is expected to provide adequate mechanical strength, controlled degradability, adhesion, and integration with the surrounding native tissue. As it does this, it mimics natural ECMs functions, which allow for nutrient diffusion and the promotion of cell survival and differentiation [7,8]. It is anticipated that the development of such an effective biomaterial would significantly enhance the potential to develop effective therapies for tissue regeneration and function improvement. One of these promising regenerative therapies is the use of in situ-forming (injectable) hydrogels: a three-dimensional (3D) scaffold that mimics the hydrated environment of articular cartilage and facilitates cell proliferation, differentiation and matrix production by using encapsulated cells [9]. Injectable hydrogels enable a perfect match with irregular cartilage defects and a proper alignment with the surrounding tissues [9,10]. Meanwhile, from the clinical point of view, implantation surgery can be avoided and replaced by a simple, minimally invasive injection [9]. Moreover, bioactive molecules or cultured cells can simply be mixed into the hydrogel precursors prior to injection [11]. Therefore, they are promising materials that can function as scaffolds for chondrocyte culture and cartilage regeneration. 

The development of injectable hydrogels as scaffolds for cartilage tissue engineering must meet certain essential conditions: biocompatibility, biodegradability, biofunctionality, and suitable mechanical strength. In our earlier studies, we developed an injectable hybrid hydrogel composed of a hyaluronic acid (HA) backbone with tyramine conjugated dextran (Dex–TA) sidechains [12]. The hydrogel gelates in situ via a biocompatible, enzymatic crosslinking reaction that forms covalent TA–TA bonds and has been shown to support the survival and growth of incorporated chondrocytes and mesenchymal stem cells as well as the deposition of a new matrix in vitro [13,14]. Using a similar mechanism, we also showed efficient gel formation after mixing HA–TA and Dex–TA conjugates [15]. The advantage of these hybrid, injectable hydrogels is that multiple functionalities can be included in one gel system to fine-tune physical properties, proteolytic degradation, and extracellular matrix production. Moreover, these hydrogels can be tailored for stiffness and degradation rate by varying polymer concentrations and ratios. In this study, we determined the optimal concentration and ratio for cell growth and matrix formation in HA–TA and Dex–TA hybrid hydrogels. To obtain these results, hydrogels at different conjugate concentrations and ratios were laden with bovine chondrocytes (bCHs), and the cartilaginous specific matrix formed in the cell/gel constructs over time was analyzed. Furthermore, physical properties like storage moduli and morphology of the hydrogels were examined.

## 2. Materials and Methods

### 2.1. Materials

Dextran (40 kDa, pharmaceutical grade) was purchased from Pharmacosmos, Holbæk. Denmark. Sodium hyaluronate (27 kDa, pharmaceutical grade) was purchased from Contipro Pharma, Dolní Dobrouč, Czech Republic. Tyramine (99%), DMF (anhydrous, 99.8%), LiCl (99.0%), p-nitrophenyl chloroformate (PNC, 96%), pyridine (anhydrous, 99.8%), DMSO-d6 (99.9%), NaCl (≥99.0%), D_2_O (99.9 atom % D), horseradish peroxidase (HRP, 325 units/mg solid) and hydrogen peroxide (30%) were purchased from Sigma-Aldrich, St. Louis, MO, USA. Tyramine HCl salt (99%) was obtained from Acros Organics, Fair Lawn, NJ, USA. 4-(4,6-Dimethoxy-1,3,5-triazin-2-yl)-4-methylmorpholinium chloride (DMTMM, 97%) was purchased from Fluorochem Ltd., Hadfield, UK. Ethanol (≥99.9%) and diethyl ether (≥99.7%) were purchased from Merck, Kenilworth, NJ, USA. Milli-Q water was used from the Milli-Q Advantage A10 system equipped with a 0.22 μm Millipak^®^-40 Express filter.

### 2.2. Synthesis of Dextran-Tyramine and Hyaluronic Acid-Tyramine

Dextran–tyramine was synthesized by the activation of dextran with PNC and subsequent aminolysis with tyramine adapted from Ramirez et al. [16]. Hyaluronic acid–-tyramine was prepared by amidation of the HA carboxyl groups with tyramine by using a procedure adapted from Rydergren [17] and D’Este et al. [18]. Detailed description of polymers synthesis can be found in the Supplementary Methods. Synthesis and characterization of Dex–TA and HA–TA polymers are described in Supplementary Figure S1. The Dex–TA used in this study had a substitution degree of 10%; i.e., 10% of the monosaccharides of dextran was modified. HA–TA had a substitution degree of 10%; i.e., 10% of the carboxylic acid groups of hyaluronic acid was modified.

### 2.3. Cell Culture and Expansion

Bovine chondrocytes (bCHs) were isolated from cartilage knee biopsies of full thickness from six-month old female calves according to the previously reported protocol [19]. bCHs were expanded in a chondrocyte proliferation medium (Dulbecco’s modified Eagle’s medium (DMEM; Gibco, Billings, MT, USA) and supplemented by 10% fetal bovine serum (FBS; Gibco), 0.2 mM ascorbic acid 2-phosphate (Sigma), 0.4 mM proline (Sigma), 1x nonessential amino acids (Gibco), 100 U/mL penicillin, and 100 µg/mL streptomycin (Invitrogen, Carlsbad, CA, USA)). The medium was refreshed twice a week, and cells were used for experiments at passage 3.

### 2.4. Hydrogel Formation

To prepare identical hydrogel samples, we designed a mold (Supplementary Figure S2). In brief, the hydrogels were prepared in a PTFE mold to produce six identical hydrogels 8 mm wide and 1.5 mm high. After dissolving the tyramine-conjugated polymers in sterile phosphate buffered saline (PBS), the polymer solution with horseradish peroxidase (HRP, 40 units/mL; Sigma-Aldrich, St. Louis, MO, USA) was incubated overnight at 4 °C on a rollerbank. The mixture was then combined with bCHs in a concentration of 10 million cells/mL. Cell-free controls were also prepared. Freshly prepared hydrogen peroxide (H_2_O_2_) was added to the mixture and immediately transferred to the mold using a 1 mL pipette after a brief vortex. The final gel concentrations were a 10% *w*/*v* or 5% *w*/*v* polymer, 10 million/mL bCHs, 4 U/mL HRP and 0.03% H_2_O_2_ (for the 10% *w*/*v* polymer) or 0.015% H_2_O_2_ (for the 5% *w*/*v* polymer). HA–TA and Dex–TA were combined in 5 ratios (100:0, 75:25, 50:50, 25:75, and 0:100), and were represented by groups A, B, C, D, and E respectively.

### 2.5. Hydrogel Incubation

After gelation, the gels were transferred to six-well plates and incubated in chondrogenic differentiation medium (DMEM supplemented with 0.2 mM ascorbic acid 2-phosphate (Sigma), 0.4 mM proline (Sigma), 100 U/mL penicillin, and 100 µg/mL streptomycin (Invitrogen), 0.1 µM dexamethasone (Sigma), 100 µg/mL sodium pyruvate (Sigma), 50 µg/mL insulin–transferrin–selenite (ITS; Sigma), 10 ng/mL transforming growth factor β-3 (TGF-β3; R&D Systems)). The medium was refreshed three times every week, and the gels were harvested at time points day 0, 7, and 21.

### 2.6. Rheological Analysis

Rheological experiments were carried out using an MCR301 rheometer (Anton Paar, Oosterhout, Nederland) using parallel plates (8 mm diameter) at 20 °C under a 0.05 N normal force in the oscillatory mode with 0.5% strain and 1.0 Hz, which was in the LVE range according to the measured frequency and strain sweeps. The cylindrical hydrogels were prepared in 8 mm wide, 1.5 mm high molds and measured after equilibrating overnight in medium. At least three specimens were tested for each sample.

### 2.7. Hydrogel Swelling Ratio

The swelling ratio was based on the weight of the hydrogel samples:

(1) $$Swelling\;ratio=\frac{w_{wet}-w_{dry}}{w_{dry}}$$

To assess swelling, the hydrogels were measured after equilibrating overnight in medium and compared to their dry weight. At least three specimens were tested for each composition.

### 2.8. Compression Tests

Compression testing was performed on the cylindrical gels as prepared and equilibrated for the rheological testing using a Texture Analyser TA–HD plus (StableMicro Systems Ltd., Surrey, UK) fitted with a 50 kg load cell. The hydrogels underwent three compression cycles with a maximum strain of 50% using a compression speed of 0.05 mm/s. The compression tests were conducted at room temperature, and at least three specimens were tested for each sample.

The following data were derived from the stress–strain curves. Maximum stress is the force needed to compress the sample until 50% strain is reached. The high strain compressive modulus was calculated from the stress–strain curves using a linear slope at a strain ranging from 40 to 49.5%. The percentage of energy dissipated during a compression–relaxation cycle was calculated by dividing the surface of the hysteresis loop by the surface under the compression trace.

### 2.9. Histology and Immunohistochemistry Staining

The samples were fixed in 10% formalin and then incubated in OCT (Thermo-scientific, Waltham, MA, USA) overnight at 4 °C. The samples embedded in OCT were then snap frozen using liquid nitrogen. Cryosections of 10 µm were cut using cryotome (Leica, Wetzlar, Germany, CM1100) and stained for sulfated glycosaminoglycan (GAG) with Alcian blue and Safranin O staining. For immunohistochemistry, cryosections were incubated with 0.3% H_2_O_2_ and blocked in 5% bovine serum albumin. Slides were subsequently incubated overnight at 4 °C with a rabbit polyclonal antibody against COL II (Abcam, Cambridge, UK). The sections were then incubated with a polyclonal goat–anti-rabbit HRP-conjugated secondary antibody (Dako, Glostrup, Denmark), followed by development with the DAB Substrate kit (Abcam). Counterstaining was performed with hematoxylin. Non-immune controls underwent the same procedure without primary antibody incubation. Both histology and immunohistochemistry stained slides were scanned with the NanoZoomer 2.0-RS slide scanner (Hamamatsu, Sendai City, Japan).

### 2.10. Live-Dead Staining

The effect of the hydrogel’s composition on cell viability was studied using a live–dead assay. At day 0 and 21, the hydrogel constructs were rinsed with PBS and stained with calcein AM/ethidium homodimer using the live–dead assay Kit (Invitrogen), according to the manufacturer’s instructions. Hydrogel/cell constructs were visualized using fluorescence microscopy (Leica DM IRB) and different areas were randomly selected. As a result, living cells fluoresce green and the nuclei of dead cells red. Image J software was used for cell counting. The cell viability was calculated by the percentage of live cells (green) in the total cells (green + red) from each area. Values represent the mean +/− standard deviation of at least 3 biological replicates.

### 2.11. RNA Isolation and Quantitative Polymerase Chain Reaction

The 5% *w*/*v* hydrogels were prepared for species-specific quantitative polymerase chain reaction (qPCR) analysis. At day 0 and 21, hydrogel samples were first homogenized by gentleMACS Dissociator according to the manufacturer’s instructions (Miltenyi Biotec). Total RNA was then isolated using the TRIzol Reagent (Ambion) according to the manufacturer’s protocol and reverse-transcribed into cDNA using the iScript cDNA Synthesis kit (Bio-Rad). A qPCR test was performed on cDNA samples by using the SensiMix SYBR& Fluorescein Kit (Bio-Rad). PCR reactions were carried out on CFX Connect™ Real-Time PCR Detection System (Bio-Rad). The cDNA was denatured at 95 °C for 10 min followed by 40 cycles. Each cycle consisted of following conditions: 15 s at 95 °C, 15 s at 60 °C, and 30 s at 72 °C. The sequence of primers for qPCR are listed in Table 1. The expression level of aggrecan (ACAN), collagen type I, II and IX (COL1, COL2, and COL9) and Osteopontin (OPN) were investigated.

### 2.12. Statistical Analysis

Data were presented as mean ± standard deviation. Statistical significance between the two groups was analyzed using a Student’s t-test. For three or more groups, a statistical comparison was done using the one-way Analysis of Variance (ANOVA) with Tukey’s post hoc analysis. A *p*-value of < 0.05 was considered statistically significant.

## 3. Results

### 3.1. Hydrogel Formation and Morphology

In this study, the hydrogels were formed by dissolving the functionalized polymers (Dex–TA DS 10% and HA–TA DS 10%) and HRP in PBS (mixed with cells where desired) and adding minute quantities of H_2_O_2_ as an oxidizing agent. Upon the addition of H_2_O_2_, the crosslinking of tyramine was initiated. Precooling the solutions on ice and using a final concentration of 4.0 U/mL, HRP gave us a working time (i.e., gelation time) of about 30 s, after which the solution gelated in the mold. The quantity of H_2_O_2_ was adapted to the molar amount of tyramine groups, ensuring complete consumption of the oxidizing agent in all conditions within the different weight concentrations. The overview figures of the hydrogels (shown in Supplementary Figure S3) reveal that the size of the hydrogels decreased after the dextran concentration was increased from 0 to 100%. Both the hydrogels with and without cells showed the same trend; however, the addition of cells increased the gel size.

### 3.2. Cell Viability of Chondrocytes in Different Hydrogels

**Figure 1:**
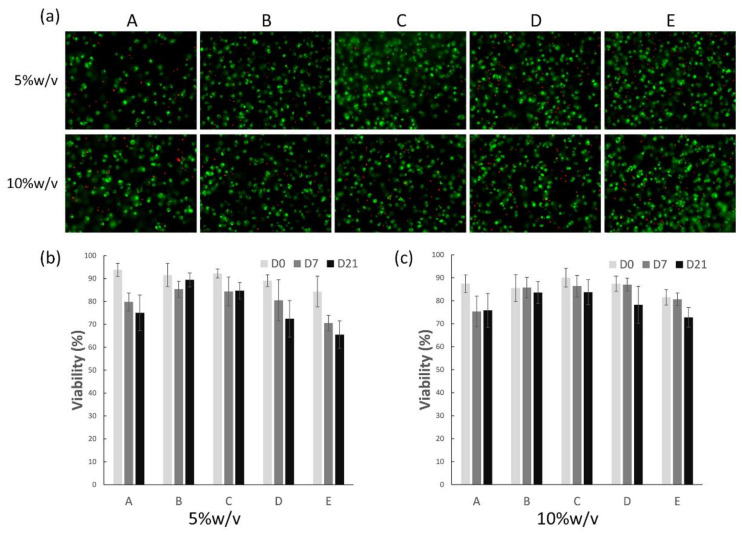
Representative figures show live–dead staining of 5 and 10% *w*/*v* hydrogels encapsulated with chondrocytes after culturing for 21 days in the chondrogenic medium (**a**). Conditions A to E represent different mix ratios of HA and Dex (100:0, 75:25, 50:50, 25:75, 0:100). Cell viability was quantified based on the live–dead staining figures of 5% *w*/*v* (**b**) and 10% *w*/*v* (**c**) hydrogels at day 0, 7 and 21.

Cell viability of the bovine chondrocytes in the hydrogels was evaluated using a live–dead assay, in which living cells were stained green and dead cells stained red (Figure 1a). We counted the amount of live and dead cells and calculated the percentage of live cells (Figure 1b,c). The results showed that the chondrocytes were distributed homogeneously inside hydrogels, and over 90% of the cells remained viable in most conditions at day 0. After 21 days of culturing in the chondrogenic medium, as shown in the figure, chondrocytes maintained their characteristic round shapes, while cell viability decreased in all conditions over time. Groups B and C (75:25 and 50:50—HA–TA and Dex–TA, respectively) showed greater cell viability than under other conditions in both 5 and 10% *w*/*v* hydrogels. Meanwhile, groups D and E, which had a higher dextran concentration, presented lower cell viability in 5% *w*/*v* conditions compared to that in 10% *w*/*v*, especially in pure dextran hydrogels (group E). On the other hand, the other groups showed similar cell viability between 5 and 10% *w*/*v* hydrogels. However, after 21 days, there was still around 70 to 90% cell survival, indicating that these biomimetic hydrogels could provide a supportive environment for chondrocyte proliferation and differentiation as well as matrix deposition.

### 3.3. Mechanical Properties

**Figure 2:**
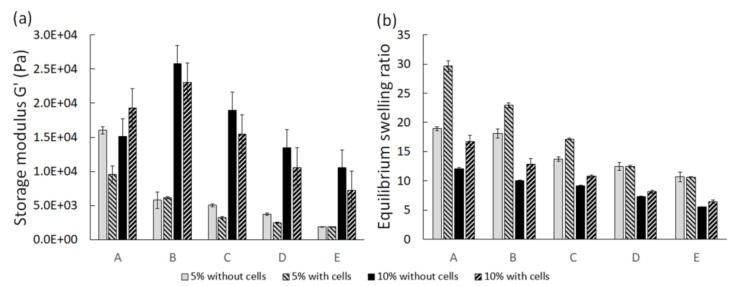
Rheological properties (**a**) and equilibrium swelling (**b**) of the hydrogels on day 0. A to E represent different HA-to-Dex ratios (100:0, 75:25, 50:50, 25:75, 0:100).

The rheological properties and equilibrium swelling of the hydrogels on day 0 are shown in Figure 2. As expected, increasing the polymer concentration of the hydrogels increased the storage modulus. Similarly, the equilibrium swelling ratio of the constructs with a lower polymer concentration was higher than for those with higher polymer concentrations. The inclusion of cells in our hydrogel constructs generally decreased the storage modulus and increased the swelling of the hydrogels, suggesting the decrease of crosslink density for these constructs. The average decrease in crosslink density was calculated to be 40% upon inclusion of cells. The calculations were based on the classical rubber elasticity theory and are provided in the Supplementary information (Table S1 and Equation (S1)).

**Figure 3:**
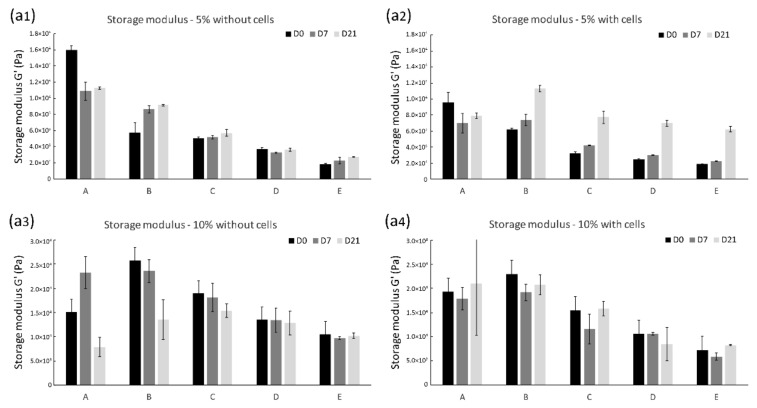

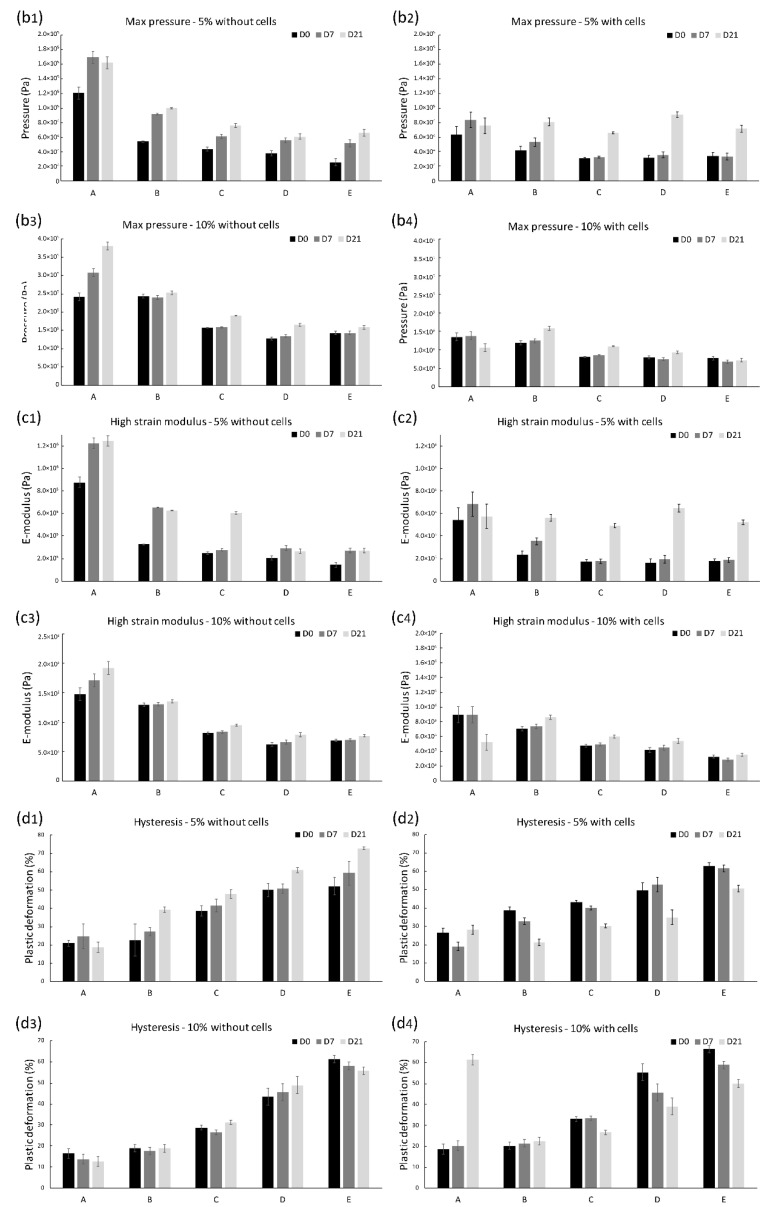
Mechanical properties of the hydrogels after 0, 7, and 21 days of culture. Conditions A to E represent different ratios of HA and Dex (100:0, 75:25, 50:50, 25:75, 0:100). (**a1**–**a4**) the storage modulus of the hydrogels, (**b1**–**b4**) the pressure needed for 50% deformation of the hydrogels, (**c1**–**c4**) the high strain Young’s modulus of the hydrogels, (**d1**–**d4**) the elasticity of hydrogels.

The mechanical properties of the hydrogels were measured after 7 and 21 days of incubation in the chondrogenic differentiation medium. Although the hydrogels with cells were weaker, upon culturing they became stronger and more elastic. After 21 days, we saw an increase in the storage modulus measured by rheology, which indicated that the hydrogel could store more deformation energy in an elastic manner that it could on day 0 and day 7. It would indicate an increase in network density, which is related to the deposition of cartilage ECM proteins as confirmed by histology. This increase in the storage modulus was most prominent for 5% *w*/*v* hydrogels, indicating that the weaker, more open structure of the hydrogel was preferred for the deposition of the cartilaginous matrix (Figure 3a1–a4). This observation was confirmed by texture analysis showing that the maximum pressure needed to compress the hydrogels to 50% strain (Figure 3b1–b4) and the E-modulus under high strain (Figure 3c1–c4) were increased in the 5% *w*/*v* hydrogels. Next to that, the elasticity of the hydrogels was also increased after 21 days, which we derived by the hysteresis in the stress-strain curves recorded (Figure 3d1–d4). 

### 3.4. Higher Dextran Concentration of Hydrogels Enhanced Matrix Deposition

GAG production by chondrocytes in all conditions was examined by histology using Safranin O staining (Supplementary Figure S4, but only Day 21 Safranin O staining results are shown). The staining results of HA hydrogel without cells showed that pure HA gel is stained by the dyes as expected (data not shown), which indicate that histology staining is not specific enough to distinguish the matrix production in these hydrogels. Nonetheless, histological staining confirmed that the chondrocytes incorporated in these hydrogels produced abundant ECM rich in GAGs after 21 days, as confirmed by the dense GAG staining in these gels. These results demonstrated that the incorporation of HA would improve the performance of Dex–TA gels in cartilage tissue engineering and that 5% *w*/*v* hydrogels showed better matrix production than did 10% *w*/*v* hydrogels.

**Figure 4:**
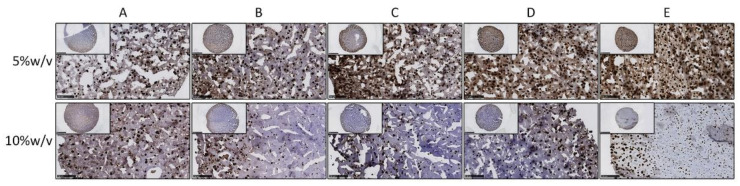
Immunohistochemistry staining of collagen type II for 5% *w*/*v* and 10% *w*/*v* hydrogels encapsulated with chondrocytes after culturing for 21 days in chondrogenic medium. From condition (**A**–**E**) represent the different conditions with different mix ratio of HA and Dex (100:0, 75:25, 50:50, 25:75, 0:100). Inserts indicate the overview of each hydrogel; scale bar = 2.5 mm. Pictures show the magnified view of each hydrogel; scale bar = 250 µm.

Collagen type II is the primary type of collagen present in articular cartilage [20]. Consequently, we performed immunohistochemistry staining to detect the specific production of collagen type II (Figure 4 and Supplementary Figure S5). After 21 days, the cartilage matrix was deposited inside different hydrogel conditions. Moreover, it clearly showed that 5% *w*/*v* hybrid hydrogels encapsulated with chondrocytes exhibited greater deposition of type II collagen than the 10% *w*/*v* groups did. Besides, the condition that was composed of 25% HA and 75% dextran (condition D) displayed the most intense staining of all the 5% *w*/*v* hybrid hydrogels. The histochemical analysis also revealed that the cartilage matrix formation was more dominant at the periphery of the hydrogels, especially in hydrogels of the 10% *w*/*v* groups.

**Figure 5:**
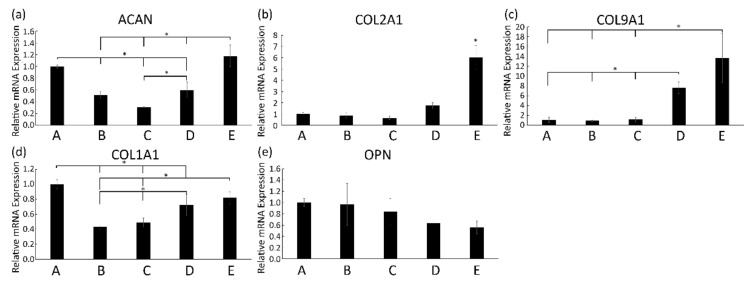
Relative mRNA expression levels for ACAN (**a**), COL2A1 (**b**), COL9A1 (**c**), COL1A1 (**d**), and OPN (**e**), expressed by bovine chondrocytes incorporated into 5% *w*/*v* hydrogels, after 21 days in culturing in the chondrogenic medium. Assignments A to E represent the different conditions with different HA-to-Dex ratio (100:0, 75:25, 50:50, 25:75, 0:100). Error bars reflect SD and * represents *p* < 0.05 compared to other indicated conditions.

Next, we performed qPCR to study the mRNA expression of chondrogenic genes in 5% *w*/*v* hydrogels combined with bCHs that were cultured in the chondrogenic differentiation medium for 21 days. The histochemical results were corroborated by gene expression analysis (Figure 5). The relative fold expression of chondrogenic related genes such as ACAN, COL2A1, and COL9A1 was up-regulated in hydrogels containing higher dextran concentrations (Figure 5a–c). The expression of SOX9 was also measured in this study, which showed a similar trend as these genes (data not shown). However, the overall mRNA expression level of SOX9 remained low in all conditions. Additionally, a decrease in the expression of COL1A1 and OPN in conditions with a higher dextran content was observed compared to the pure HA group (Figure 5d,e).

## 4. Discussion

In this work, different ratios of HA and dextran-based hybrid hydrogels at both 10% *w*/*v* and 5% *w*/*v* were prepared with a mold to determine physical property changes and their effects on the cellular behavior and cartilage matrix formation. Our results indicated that the incorporation of chondrocytes in the hydrogels introduced soft pockets in the hydrogel matrix, which decreased their mechanical properties on a macroscopic level. Interestingly, the rheological and compression analysis indicated that 5% *w*/*v* hydrogels laden with cells showed a significant increase in mechanical properties after culturing for 21 days. Moreover, compared to 10% *w*/*v* hydrogels, the 5% *w*/*v* hybrid hydrogels showed enhanced deposition of cartilage matrix, especially in the constructs with higher Dex–TA concentrations.

The chondrocytes encapsulated inside hydrogels retained a round shape at 21 days in culture. However, compared to previously reported HA-g-Dex–TA hydrogels, hydrogels in this study showed decreased cell viability [12], which can be firstly explained by the difference in measured time points. Initially, over 90% of the incorporated cells were alive, demonstrating the cytocompatibility of the hybrid hydrogels. Decreased cell viability after 21 days could also be explained by the procedure of hydrogel formation. To make sure all components were incorporated homogeneously, the solution was mixed by vortexing after the addition of H_2_O_2_. The shear forces during vortexing could damage the incorporated cells. Further studies need to address the force effect on the cells and determine the proper vortexing speed. Moreover, the relatively low cell viability observed for the Dex–TA hydrogel may be attributed to the increasing crosslinking density of the constructs. The limited exchange of nutrients and waste products to the surrounding culture media can reduce cell viability [21].

With the help of the mold we designed, highly controlled cylindrical hydrogels were formed by dissolving the polymers and HRP in PBS and adding H_2_O_2_ as an oxidizing agent. Compared to the pure hydrogels without cells, the inclusion of cells increased hydrogel swelling, which could have indicated a decrease in crosslink density for these constructs. The increased size of hydrogels with higher HA concentration can be explained by an increase in water uptake resulting from the electrostatic repulsion of negatively charged HA chains at pH 7.4 [12]. This swelling behavior also suggested a decrease in network density as a result of degradation [22]. HA is an essential component of the ECM in cartilage tissue, which is biodegradable via enzymatic hydrolysis using hyaluronidase (HAse) [23,24]. HA degraded by the HAse expressed by the incorporated chondrocytes in the hybrid hydrogels could have been another reason for this behavior [25].

In the design of hydrogels as scaffolds for cartilage repair, adequate mechanical support is a critical requirement. The scaffold should be mechanically stable in order to protect the seeded cells and the developing tissue and to withstand the physiological load [26]. On a cellular scale, the mechanical properties of a scaffold are potent regulators of cell migration and their phenotypes [27]. The mechanical properties of the studied hydrogels were adjusted by varying the ratio of dextran and hyaluronic acid and the polymer concentration. The evaluation of these properties is an essential parameter in predicting the possibility of tissue production and construct quality. Rheological studies on the constructs were performed to determine storage and loss moduli, which are values for elasticity and viscosity, respectively [28]. Compression of cylindrical hydrogel samples between two plates yields a stress–strain curve, from which the elastic modulus and other mechanical properties can be derived [29]. Therefore, physical properties were determined by rheology and texture analysis at different time points to investigate how gel composition and mechanical properties could influence cell behavior and how the cells consequently would influence hydrogel characteristics.

In line with the previous report, increasing the polymer concentration in the hydrogels increased the storage modulus because hydrogels prepared at a concentration of 10% *w*/*v* showed a higher storage modulus than the 5% *w*/*v* hydrogels. Furthermore, by encapsulating the chondrocytes, the corresponding storage modulus G’ values decreased, suggesting a decrease of crosslink density of these constructs.

However, upon culturing in a differentiation medium for 21 days, these gels became stronger and more elastic. Compared to pure gels without cells, chondrocytes laden constructs showed enhanced storage moduli after 21 days. Especially in cell laden 5% *w*/*v* hydrogels, a significantly increased storage modulus was observed in rheological measurements. This improvement was most evident in constructs with a higher Dex–TA concentration. However, the 10% *w*/*v* hydrogels showed only moderate changes compared to the initial values. This observation was confirmed by rheological analysis. Previous reports indicated that the compressive modulus for articular cartilage is 0.24 to 0.85 MPa [30] which is substantially higher than the compressive modulus, which was obtained after three weeks of culture of the hydrogel constructs. The compressive modulus of the latter constructs approached the mechanical properties of the chondron, which has a modulus of around 70 kPa [31]. This suggested that in 21 days of being cultured the chondrocytes created an environment that resembled at least some of the properties of the native chondron. These results also suggested that the increased network density in the hydrogels was related to ECM deposition and that the 5% *w*/*v* hydrogels, which are weaker and have a more open structure, were preferred for the deposition of cartilaginous matrix. Furthermore, a higher Dex–TA concentration was shown to promote this.

This hypothesis was confirmed by the cartilage matrix-related staining. Compared to day 0 constructs, abundant deposition of cartilage matrix was observed inside different hydrogel conditions after 21 days, which can partly explain the increased stiffness and elasticity of the cell-laden hydrogels. Interestingly, it was clearly shown that 5% *w*/*v* hybrid hydrogels laden with chondrocytes exhibited a denser deposition of cartilage matrix compared to the 10% *w*/*v* constructs. These data were consistent with the significantly increased mechanical properties in 5% *w*/*v* hydrogels from rheology and compression analysis. It was likely caused by a greater diffusion of nutrients and growth factors in the 5% *w*/*v* hydrogels than in the 10% *w*/*v* hydrogels since 10% *w*/*v* hydrogels showed higher mechanical strength, which is known to compromise diffusion ability [32]. In addition, a higher polymer concentration also resulted in a decreased accumulation of matrix components such as proteoglycans and collagen type II [33]. Indeed, in 10% *w*/*v* hydrogels, the formation of cartilage matrix was predominantly observed on the periphery of the hydrogels.

Moreover, 5% *w*/*v* hybrid hydrogels with a higher Dex–TA content produced an abundant, homogeneously distributed cartilage matrix, which was corroborated by the up-regulated expression of chondrogenic related genes. Considering that HA is present in native cartilage and plays a role in influencing the cell phenotype and matrix production [34,35], incorporation of HA in a hybrid hydrogel would improve its performance in cartilage tissue engineering. In conclusion, these results indicated that 5% *w*/*v* hydrogels showed better matrix production than 10% *w*/*v* hydrogels and that a combination of 25% HA and 75% Dex is probably the optimal hybrid condition for cell growth and matrix formation.

## 5. Conclusions

We prepared hybrid hydrogels of different ratios of HA and dextran of controlled size and shape at both 10% *w*/*v* and 5% *w*/*v* using a designed mold.The behavior of chondrocytes incorporated in the hybrid hydrogels demonstrated that the gel systems had proper biocompatibility. Our data demonstrated that the presence of chondrocytes decreased the hydrogels’ initial mechanical properties. Nonetheless, chondrocyte-laden constructs showed an enhanced storage modulus after 21 days. Rheological and compression analysis indicated that 5% *w*/*v* hydrogels laden with cells particularly showed a significant increase in mechanical properties. Moreover, compared to 10% *w*/*v* hydrogels, the 5% *w*/*v* hybrid hydrogels induced enhanced matrix deposition (increased glycosaminoglycan and collagen production). This observation was most evident in constructs with a higher Dex–TA concentration. Altogether, these data suggest that a 5% *w*/*v* hybrid hydrogel with 25% HA and 75% Dex is a promising construct for cartilage repair approaches.

## Figures and Tables

**Table 1:** Primers used for quantitative RT-PCR.

Gene Name	Primer Sequence	Product Size (bp)
Bovine specific GAPDH	F: 5′ GCCATCACTGCCACCCAGAA 3’	207
	R: 5′ GCGGCAGGTCAGATCCACAA 3’	
Bovine specific Aggrecan	F: 5′ GACCAGAAGCTGTGCGAGGA 3’	319
	R: 5′ GCCAGATCATCACCACACAG 3’	
Bovine specific Collagen II	F: 5′ ATCAACGGTGGCTTCCACT 3’	263
	R: 5′ TTCGTGCAGCCATCCTTCAG 3’	
Bovine specific Collagen IX	F: 5′ GGACTCAACACGGGTCCACA 3’	102
	R: 5′ ACAGGTCCAGCAGGGCTTTG 3’	
Bovine specific Collagen I	F: 5′ GCGGCTACGACTTGAGCTTC 3’	102
	R: 5′ CACGGTCACGGACCACATTG 3’	
Bovine specific Osteopontin	F: 5′ ACTGGACTCTTCTCGCCGCC 3’	90
	R: 5′ CGGAGGCAATGCCCAAGAGGC 3’	

## Data Availability

The data presented in this study are available on request from the corresponding author.

## Supplementary Materials

The following are available online at https://www.mdpi.com/article/10.3390/polym13091526/s1, synthetic methods, Figure S1: 1H NMR spectra of dextran and hyaluronic acid tyramine conjugates (Dex–TA and HA-TA), Figure S2: Sketches of the prepared hydrogel molds, Figure S3: Overview morphology of 5% *w*/*v* and 10% *w*/*v* hydrogels, Figure S4: Safranin O staining of 5% *w*/*v* and 10% *w*/*v* hydrogels, Figure S5: Immunohistochemistry staining of collagen type II for 5% *w*/*v* hydrogels. Table S1: Effective crosslink density, $\nu_{e}$ (mM). Equation (S1): Crosslinking density.
